# Post-traumatic trigeminal neuropathy: correlation between objective and subjective assessments and a prediction model for neurosensory recovery

**DOI:** 10.1186/s10194-021-01261-3

**Published:** 2021-05-24

**Authors:** Jeroen Meewis, Tara Renton, Reinhilde Jacobs, Constantinus Politis, Fréderic Van der Cruyssen

**Affiliations:** 1 Department of Oral and Maxillofacial Surgery, University Hospitals Leuven, Kapucijnenvoer 33, 3000 Leuven, Belgium; 2 OMFS-IMPATH Research Group, Department of Imaging and Pathology, Faculty of Medicine, University Leuven, Leuven, Belgium; 3 Department of Oral Surgery, King’s College London Dental Institute, London, UK; 4 Department of Dental Medicine, Karolinska institutet, Stockholm, Sweden

**Keywords:** Post-traumatic trigeminal neuropathy (PTN), Trigeminal nerve, Neurosensory test, Subjective well-being, Quality of life, Neurosensory recovery

## Abstract

**Background:**

Post-traumatic trigeminal neuropathy (PTN) can have a substantial effect on patient well-being. However, the relation between the neuropathic symptoms and their effect on psychosocial functioning remains a matter of debate. The purpose of this study was to evaluate the association between objective and subjective assessments of neurosensory function in PTN and predict neurosensory outcome using baseline measurements.

**Methods:**

This prospective observational cohort study included patients diagnosed with PTN at the Department of Oral and Maxillofacial Surgery, University Hospital Leuven, Belgium, between April 2018 and May 2020. Standardized objective and subjective neurosensory examinations were recorded simultaneously on multiple occasions during the follow-up period. Correlation analyses and principal component analysis were conducted, and a prediction model of neurosensory recovery was developed.

**Results:**

Quality of life correlated significantly (*P* < 0.05) with percentage of affected dermatome (ρ = − 0.35), the presence of brush stroke allodynia (ρ = − 0.24), gain-of-function sensory phenotype (ρ = − 0.41), Medical Research Council Scale (ρ = 0.36), and Sunderland classification (ρ = − 0.21). Quality of life was not significantly correlated (*P* > 0.05) with directional discrimination, stimulus localization, two-point discrimination, or sensory loss-of-function. The prediction model showed a negative predictive value for neurosensory recovery after 6 months of 87%.

**Conclusions:**

We found a strong correlation of subjective well-being with the presence of brush stroke allodynia, thermal and/or mechanical hyperesthesia, and the size of the neuropathic area. These results suggest that positive symptoms dominate the effect on affect. In patients reporting poor subjective well-being in the absence of positive symptoms or a large neuropathic area, additional attention towards psychosocial triggers might enhance treatment outcome. The prediction model could contribute to establishing realistic expectations about the likelihood of neurosensory recovery but remains to be validated in future studies.

**Supplementary Information:**

The online version contains supplementary material available at 10.1186/s10194-021-01261-3.

## Background

Post-traumatic trigeminal neuropathy (PTN) [1] is a well-known complication in the oral and maxillofacial field. Many procedures may lead to iatrogenic lesions of the trigeminal nerve, and 45%–70% of PTN arises from the removal of third molars [2, 3]. Other procedures include local anesthetic injection, dental implant surgery, endodontic treatment, and several other interventions [3–12]. There is a dominant representation of lingual nerve (LN) and inferior alveolar nerve (IAN) injuries, accounting for up to 90% of all cases of PTN [2, 3, 13]. In major maxillofacial or tumor ablation surgery, these injuries are often a calculated risk. However, in all other cases, the postoperative presence of permanent neurosensory impairment is unexpected. Fortunately, 90% of these injuries are temporary and subside within 8 weeks [4, 9].

Nevertheless, PTN can interfere with a wide variety of social functions and daily activities such as eating and drinking, shaving, kissing, tooth brushing, and applying make-up [14]. In addition, PTN can lead to a substantial psychosocial and affective burden, particularly in patients who experience severe neuropathic pain as part of the condition [15]. In these cases, the more specific term “post-traumatic trigeminal neuropathic pain” is used, as described in the recently introduced International Classification of Orofacial Pain (ICOP) criteria [1]. In our study, we use the umbrella term PTN to describe either a painful or a non-painful PTN. Robert et al. [16] reported that 78% of oral and maxillofacial surgeons will be involved in one or more cases of permanent IAN injury and 46% in one or more instances of permanent LN injury over their practice lifetimes. Therefore, every oral and maxillofacial surgeon should understand the proper prevention, prediction, and management of PTN because failing to do so can lead to significant patient distress and often trigger litigation [17, 18].

To date, consensus is lacking regarding which therapy or timing is best. Different surgical procedures have been applied with varying success [19, 20]. A re-intervention carries the risk of escalating neuropathic symptoms, and the consequence is that 33% of patients decline reparative surgery when offered [21]. In addition, patients with PTN have mixed responses to medications, which all have significant side effects. Therefore, the outcome of PTN treatment is largely disappointing, leaving both patient and doctor frustrated. All interventions are targeted to improving quality of life through pain reduction, sensory improvement, functional recovery, the development of efficient coping strategies, or a combination of these outcomes. Some patients show limited symptoms yet still report a poor quality of life, whereas others experience a relatively high degree of physical impairment but seem to cope well.

Although reports have described objective neurosensory functioning and subjective well-being in PTN, few studies have evaluated the correlation between these objective and subjective measurements. Here, we sought to answer the following three questions: Is there a correlation between the objective and subjective measurements? Which of these objective measurements has the greatest correlation with subjective well-being? Can we predict neurosensory outcome using baseline measurements?

## Methods

This study is reported in accordance with the EQUATOR guidelines (Enhancing the Quality and Transparency of Health Research) and STROBE agreement (Strengthening the Reporting of Observational Studies in Epidemiology). Ethical approval was obtained from the Ethics Committee of the University Hospital Leuven (S61077, B322201835541). It was performed in accordance with Good Clinical Practice standards and the Declaration of Helsinki.

### Patient selection

This prospective observational study included 46 patients (16 men, 30 women) who were diagnosed with PTN at the Department of Oral and Maxillofacial Surgery, University Hospital Leuven, Belgium, between April 2018 and May 2020. Whenever ICOP [1] diagnostic criteria for PTN were met, patients were seen for a neurosensory consultation at our department by one investigator (FVDC). After patients gave informed consent, baseline and follow-up for both objective and subjective assessment of neurosensory function were performed by FVDC, as described below. Case-wise deletion was used to ensure a true correlation matrix.

### Data collection

#### Objective assessment

Neurosensory testing started with delineating and photographing the neuropathic zone. With this approach, both the patient and practitioner can review the digital photograph, which can then be added to the patient’s file. We used this image to describe a percentage of the affected dermatome as well as to visualize its evolution. For this purpose, the reverse end of an anesthetic needle was moved across the surface from the unaffected to affected area [22, 23]. Then, two-point discrimination, stimulus localization, and directional discrimination were examined using a light brush technique, along with response to hot and cold stimuli, all based on previously described methods [3, 22, 24, 25]. When applicable, the presence of brush stroke allodynia was noted separately. A Medical Research Council Scale (MRCS) score for sensory recovery (Supplemental Table S1) [26] was recorded, and a Sunderland clinical rating scale was used (Miloro modification, Supplemental Figure S1). Based on these findings, a code for sensory phenotype was assigned to each individual. All codes consist of a letter L (loss-of-function or sensory deficit) and a letter G (gain-of-function or hyperesthesia), followed by number 0 (none), 1 (thermal), 2 (mechanical), or 3 (mixed). For example, L3G0 indicated a patient with mixed sensory loss and no mechanical or thermal hyperesthesia. Depending on the indication, quantitative sensory testing was performed according to the German Research Network on Neuropathic Pain protocol [27, 28], as well as magnetic resonance neurography (MRN), according to the institutional protocol [29].

#### Subjective assessment

Subjective measurements consisted of several questionnaires completed during each follow-up visit or afterwards by mail or telephone. These questionnaires are the EuroQol five-dimension scale (EQ5D-5 L), General Anxiety Disorder 7 (GAD-7), Patient Health Questionnaires (PHQ) 9 and 15, Douleur Neuropathique 4 (DN4), and the Brief Pain Inventory (BPI). Pain was assessed on a visual analogue scale (VAS; ranging from 0 to 100).

The EQ5D-5 L assesses five domains on a five-point ordinary scale. The domains are mobility, self-care, usual activities, pain/discomfort, and anxiety/depression. A score of 0 indicates no problems at all in a domain, 1 indicates slight problems, 2 suggests moderate problems, 3 indicates severe problems, and 4 indicates extreme problems. Patients self-rated their health on the VAS from 0 (worst) to 100 (best health they could imagine).

The PHQ-9 questionnaire consists of nine questions about the severity of depressive complaints based on the DSM-IV criteria. Each question is scored from 0 (not at all) to 3 (almost daily), resulting in a total score of 0 to 27 points. Score ranges are 0–4 for no/minimal depression, 5–9 for mild depression, 10–14 for moderate depression, 15–19 for moderately severe depression, and 20 or greater for severe depression.

Symptoms of anxiety were assessed using the GAD-7 questionnaire. The score is calculated in the same way as the PHQ-9 questionnaire, using response scores of 0 (not at all), 1 (several days), 2 (more than half the days), or 3 (nearly every day), which are added together for the seven questions. Cutoffs are a score of 5 for mild anxiety, 10 for moderate anxiety, and 15 for severe anxiety.

The PHQ-15 is a self-administered version of the PRIME-MD diagnostic used for the detection of patients at risk for somatoform disorders. The PHQ-15 covers 15 somatic symptoms of the PHQ, with each one scored from 0 (no symptoms of … at all) to 2 (a lot of symptoms of …). Cutoffs are scores of 5 for low somatic symptom severity, 10 for medium severity, and 15 for high severity.

Pain quality was assessed using the seven yes/no questions of the DN4. Patients were asked if the pain had the characteristics of burning, painful cold, or electrical shocks and whether the pain was accompanied by a tingling, stinging, numbness, or itching sensation in the same area. A point is given for every positive answer (maximum, 7 points), and a score of 3 or greater supports a diagnosis of neuropathic pain.

Pain intensity and pain interference in activity were assessed using the BPI questionnaire, measuring pain intensity in four categories (worst, least, on average, currently) and pain interference in six categories (general activity, mood, ability to walk, normal work, social interaction, joy in life). Each category is rated on a scale from 0 to 10, with 10 indicating complete interference in the respondent’s life.

Patients were asked to score their current subjective function, ranging from 0 (complete anesthesia) to 20 (20 for the worst pain imaginable). A score of 10 indicates normal function and no deficit.

Secondary study variables collected for each patient were demographic data, signs and symptoms of the neuropathic sensation, and type of procedure associated with the injury. Possible injuries were local anesthesia, third molar removal, (ortho)gnathic surgery, implant placement, endodontic treatment, facial trauma, non-wisdom tooth extraction, or other. Additional information gathered included site of injury (branch and side) in the trigeminal distribution area, elapsed time since the traumatic event, preferred imaging modalities, selected therapy, whether or not a diagnostic test (quantitative sensory testing or MRN) was performed, and whether or not the result of any such tests affected established policy.

### Statistical analysis

All data were assessed by a certified statistician using R-statistics version 4.0.3 (The R-Foundation for Statistical Computing). Descriptive statistics were used to compare demographic data with the neurosensory test findings. Univariate relations between variables were assessed with the Pearson correlation coefficient, except when at least one of the variables was categorical. In those cases, the Spearman rank correlation coefficient was used. Principal component analysis for binary and categorical data was applied. Biplots were drawn using the loadings and scores from the principal component analysis with respect to the first two principal components. A stepwise model selection for the generalized linear model for binary data using a logit link was applied to find the combination of variables with the best relation to recovery status after 6 months.

## Results

In this prospective cohort study, from April 2018 to May 2020, 46 patients were diagnosed with PTN at our department. Nine of these patients were excluded because of missing data, and one patient declined informed consent. The remaining group of 36 patients consisted of 23 women and 13 men, with a mean age of 42 years (SD 12.5, range 23–68). Patient characteristics are shown in Supplemental Table S2. Almost all patients were referred by an oral and maxillofacial surgery specialist (*n* = 32; 89%). The remaining four patients were referred by an external dentist.

The mean duration of injury to initial clinical examination was 210 days (SD 289, range 3–1073). The average follow-up period was 566 days (SD 218, range 149–865), with an average of six follow-up visits during which objective and subjective assessments were repeated (range 3–8). In total, 199 neurosensory consultations were held.

Distribution of cases by mechanism of injury identified third molar removal as the most common, in 47% of patients (*n* = 17), followed by 11% each for implant placement and facial trauma (each, *n* = 4), 8% for local anesthesia (*n* = 3), and 6% for non-wisdom tooth extraction and endodontic treatment (*n* = 2). A total of 14% of cases were classified as “other” (*n* = 5) (Supplemental Figure S2).

The IAN was affected in 23 patients (64%), the LN in 10 (28%), the maxillary nerve in 7 (19%), and the ophthalmic nerve in one (3%). Right-sided PTN was present in 19 patients (53%), and 17 patients reported left-sided PTN (47%). No cases of bilateral involvement were detected.

Quantitative sensory tests were performed in five patients. Of seven patients in whom magnetic resonance imaging was performed, findings for five of them resulted in a change in management, including surgical reintervention in three. Microsurgery was performed in seven cases (19%). Surgical treatment was always exploratory in nature and consisted of external neurolysis, internal neurolysis, neurorrhaphy, and/or neuroma excision. No interpositional grafts were used in this series.

### Objective assessments

Mean percentage of affected dermatome was 91% (SD 21%) at baseline. At final follow-up, the mean percentage of affected dermatome decreased to 40% (SD 46%). In 11 patients (31%), the area remained identical to baseline findings, and in 16 patients (44%), neurosensory tests could no longer define an affected area. In this last group, it took an average of 253 days (median 187, SD 200) until the neuropathic zone could no longer be demarcated.

Initial two-point discrimination showed an average of 14 mm (SD 7 mm) for the affected side and 6 mm (SD 3 mm) for the unaffected side. These measurements evolved to an average final two-point discrimination of 8 mm (SD 5 mm) for the affected side in the total study population. Nine patients (25%) had an uncompromised two-point discrimination at baseline. In patients whose two-point discrimination for the affected side reached values identical to the unaffected side, the average time to that outcome was 227 days.

Eleven patients (31%) had brush stroke allodynia on initial presentation. During the follow-up period, 15 patients (42%) presented with brush stroke allodynia at least once. At the final follow-up, brush stroke allodynia remained present in five patients (14%), among whom three had it at the initial presentation and two developed it and experienced its persistence afterwards.

Stimulus localization was completely absent in 11 patients (31%) at time of initial measurements, whereas in 18 patients (50%), stimulus localization was unimpaired at baseline. At final follow-up, however, 28 patients (78%) had values similar to those in healthy individuals, with an average time to this outcome of 70 days (SD 53). Eight patients (22%) continued to experience a sub-optimal ability to locate a stimulus.

Directional discrimination showed a similar pattern: It was absent in 10 patients (28%), and 18 patients (50%) had no impairment at baseline. A total of 29 patients (81%) reached optimal final follow-up values in 81 days, on average (SD 102). Seven patients with PTN could not perfectly discriminate direction of movement at the end of the evaluation period.

Baseline and follow-up MRCS and Sunderland scores are shown in Supplemental Figure S3 and Supplemental Figure S4 respectively. At baseline, the MRCS score was S0 for five patients, S2 for one patient, S2+ for ten patients, S3 for eight patients, and S3+ for 11. One patient had a baseline MRCS score of 4. Upon study completion, 23 patients (64%) showed complete recovery (S4), seven had a score of S3+, and for one, the score was S3, for a total of eight additional patients (22%) with limited negative clinical symptoms and no residual overresponse to stimuli. The remaining five patients (14%) did not experience recovery beyond S2+ and thus continued to have positive symptomatology.

Distribution by Sunderland classification showed unimpaired level A testing (group I) in 10 patients (28%) at baseline and mildly impaired contact detection (level B testing; group II) in five patients (14%). Level C testing revealed a moderately impaired pain sensitivity in five patients (14%, group III), severely impaired in 11 patients (31%, group IV), and complete anesthesia in five patients (14%, group V). Upon study completion, 25 patients (69%) were classified into group I, 3 patients (8%) into group II, and 2 (6%) into group III, and 6 (17%) remained in group IV.

Distribution by sensory phenotype is shown in Supplemental Figure S5. Most patients began with mixed sensory loss (22 patients; 61%) and absence of hyperesthesia (19 patients; 53%). Isolated mechanical hypoesthesia was seen in 9 (25%) patients, and one patient (3%) had thermal hypoesthesia. Five patients (14%) had isolated mechanical hyperesthesia, and one (3%) had thermal hyperesthesia. Four patients (11%) showed no negative symptoms, and eleven (31%) had mixed positive symptoms at the initial presentation.

### Subjective assessments

The most reported symptom was numbness in 31 cases (86%), followed by pain in 16 cases (44%) and stinging pain in 11 (31%). Nagging, burning, sensitive, and swollen sensations were all described by 10 patients (27%). A stinging or pulling sensation was each reported by seven patients (19% each), and an electrical or tickling sensation was each mentioned by six patients (17% each) (Supplemental Table S2).

Mean QoL increased from 59/100 to 72/100 during the study period. In patients with pain as their main complaint, mean baseline Pain-VAS was 46/100 (SD 27), and mean QoL was 52/100 (SD 20). At the final follow-up, mean pain on the VAS in this group was 26/100 (SD 37), and mean QoL was 69/100 (SD 16). On initial presentation, 8 of 13 men (62%) reported pain, whereas only 8 of 23 women (35%) did so. For painful PTN, women had a mean baseline Pain-VAS of 33/100 (SD 21) and an increased final VAS score of 41/100 (SD 44). In contrast, men with painful PTN started with a mean VAS score of 57/100 (SD 28) and ended with a VAS of 15/100 (SD 28). Women with painful PTN had a mean QoL of 50/100 (SD 24) at the initial visit, which increased to 64/100 (SD 20). Men with painful PTN went from an average QoL of 54 (SD 17) to 74 (SD 13).

GAD-7 questionnaires revealed a baseline absence of anxiety in 16 patients (44%), mild anxiety in 15 patients (42%), moderate anxiety in one patient (3%), and severe anxiety in 4 patients (11%). At final follow-up, the group without anxiety increased to 22 patients (61%), mild anxiety decreased to 8 patients (22%), moderate anxiety ended with 2 patients (5%), and severe anxiety with 3 patients (8%). Three of the four patients with severe anxiety at baseline still had severe anxiety at the last follow-up. The fourth patient had moderate anxiety at the final follow-up, but with complete resolution of the neurosensory disturbances.

Results for the PHQ-9 questionnaires showed no depression in 12 individuals (33%) at initial measurement, mild depression in 15 patients (42%), moderate depression in 5 patients (14%), moderately severe depression in 2 (6%), and severe depression in 2 (6%). At the end of the study, the group without depression had grown to 20 patients (56%), mild depression had decreased to 8 patients, (22%), and moderate depression to one patient (3%). The number of patients with moderately severe depression increased to three (8%), and the number with severe depression increased to four patients (11%).

At the initial diagnosis, somatic severity of symptoms (PHQ-15) was absent in 13 (36%), low in 8 (22%), medium in 11 (31%), and high in 4 (11%) patients. After the follow-up period, symptoms were absent in 18 (50%), low in 9 (25%), medium in 7 (19%), and high in 2 (6%).

The total study population scored an average of 3/7 on the DN4 questionnaire at baseline. This value decreased over time to an average of 1/7 at the final follow-up.

Self-perceived subjective functioning is shown in Supplemental Figure S6. At baseline, 23 patients reported neurosensory loss as a primary burden, whereas 13 patients reported that their impaired functioning was mainly caused by pain complaints or other positive symptoms. As the study progressed, recurring questions concerning self-perceived functioning revealed similar trends in time and magnitude towards normal functioning, with a small number of outliers represented on both sides who did not experience a return to self-perceived normal functioning.

### Correlations

#### Objective measurements

**Fig. 1:**
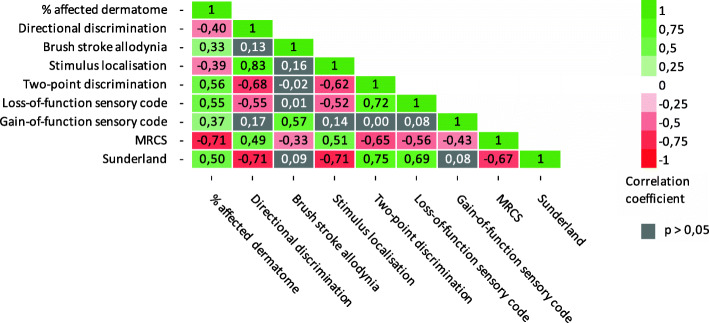
Correlation between objective neurosensory measurements. Correlation coefficients of significant positive correlations (*P* < 0.05) are shown in green. Correlation coefficients of significant negative correlations (*P* < 0.05) are shown in red. Non-significant correlations (*P* < 0.05) are displayed in grey. Neurosensory tests consisted of percentage of affected dermatome, directional discrimination, the presence of brush stroke allodynia, stimulus localization, two-point discrimination, sensory phenotype loss- and gain-of-function, MRCS, and Sunderland score

Correlations between all objective measurements are shown in Fig. 1. This figure shows that most of the objective neurosensory measurements were statistically significantly (*P* < 0.05) correlated with each other. A very strong positive correlation was seen between stimulus localization and directional discrimination (ρ = 0.83), between loss-of-function sensory code and two-point discrimination (ρ = 0.72), and between two-point discrimination and the Sunderland score (ρ = 0.75). A very strong negative correlation was seen between MRCS score and percentage of affected dermatome (ρ = − 0.71), directional discrimination and Sunderland (ρ = − 0.71), and stimulus localization and Sunderland (ρ = − 0.71). Brush stroke allodynia and gain-of-function sensory code correlated significantly (*P* < 0.05) only with percentage of affected dermatome, MRCS score, and each other.

**Fig. 2:**
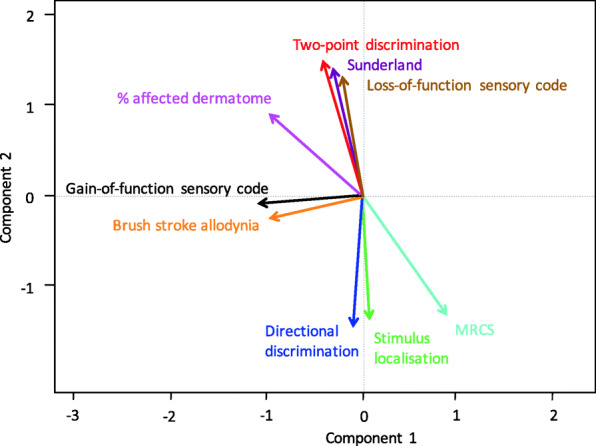
Biplot of objective neurosensory measurements. An acute angle indicates a positive correlation. A 90-degree angle indicates no correlation between the two variables, and an obtuse angle indicates a negative correlation. The more similar the direction of two vectors, the stronger the correlation between these variables. This biplot shows a strong correlation between two-point discrimination, Sunderland score, loss-of-function sensory code and percentage of affected dermatome. Also, directional discrimination, stimulus localization, and MRCS score show a strong correlation. Gain-of-function sensory code and brush stroke allodynia show a strong correlation with each other but are far less correlated with the other variables

Biplots were drawn using the loadings and scores from the principal component analysis. A biplot of all objective measurements is shown in Fig. 2. The orientation of the vectors relative to each other illustrates their correlation to one another. An acute angle between the different measurements indicates a positive correlation. A 90-degree angle implies no correlation between the two variables, and an obtuse angle signifies a negative correlation. The more similar the direction of two vectors, the stronger the correlation between the neurosensory tests. Figure 2 shows that a higher two-point discrimination, Sunderland score, and loss-of-function sensory code were strongly correlated with each other and with the percentage of affected dermatome. Their vectors almost look like the mirror image of directional discrimination, stimulus localization, and MRCS score, indicating a strong negative correlation for these factors. Gain-of-function sensory code and brush stroke allodynia showed a strong correlation with each other but were far less correlated with the other variables.

#### Subjective measurements

**Fig. 3:**
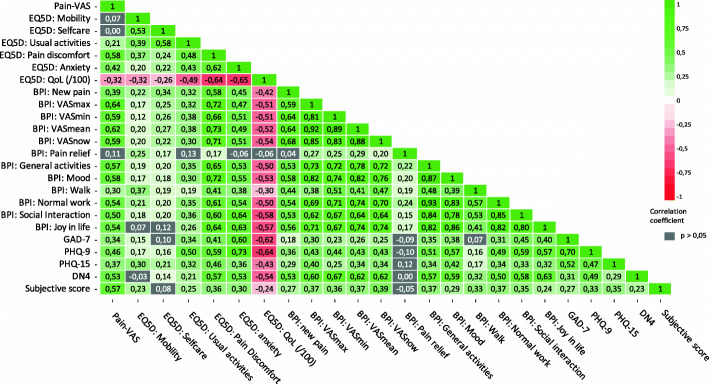
Correlation between subjective neurosensory measurements. Correlation coefficients for significant positive correlations (*P* < 0.05) are shown in green. Correlation coefficients for significant negative correlations (*P* < 0.05) are shown in red. Non-significant correlations (*P* > 0.05) are displayed in grey. The questionnaires were the pain visual analogue score (VAS) score, the EuroQol five-dimension scale (EQ5D-5 L), Brief Pain Inventory (BPI), General Anxiety Disorder 7 (GAD-7), Patient Health Questionnaire 9 and 15 (PHQ-9 and PHQ-15), Douleur Neuropathique 4 (DN4), and subjective functioning. This figure shows that most of the questionnaires were statistically significantly correlated with each other e.g. Pain-VAS correlated significantly with GAD-7, PHQ-9, PHQ-15, DN4, subjective score, and quality of life (EQ5D:QoL). Also, Quality of life showed a significant negative correlation (in red) with most questionnaire scores. GAD-7 and PHQ-9 showed the strongest negative correlation with quality of life

Correlations between all subjective measurements are shown in Fig. 3. As the figure indicates, most of the subjective neurosensory measurements were statistically significantly (*P* < 0.05) correlated with one another. Pain VAS correlated significantly with GAD-7, PHQ-9, PHQ-15, DN4, subjective score, and QoL. Also, results of the following questionnaires correlated with each other on a statistically significant level: GAD-7 with PHQ-9, PHQ-15, DN4, and subjective score; PHQ-9 with PHQ-15, DN4, and subjective score; PHQ-15 with DN4 and subjective score; and DN4 with subjective score. There was a statistically significant negative correlation between quality of life and GAD-7, PHQ-9, PHQ-15, DN4, and subjective score. Thus, a higher score on one of these questionnaires was generally associated with lower self-perceived quality of life, and the scores for PHQ-9 and GAD-7 showed the strongest correlation with quality of life. Pain relief (BPI) using a prescribed drug regimen correlated statistically significantly with EQ5D-5 L scores for pain discomfort, mobility, and self-care and with VAS max, VAS min, VAS mean, and VAS now, but not with the other questionnaires.

**Fig. 4:**
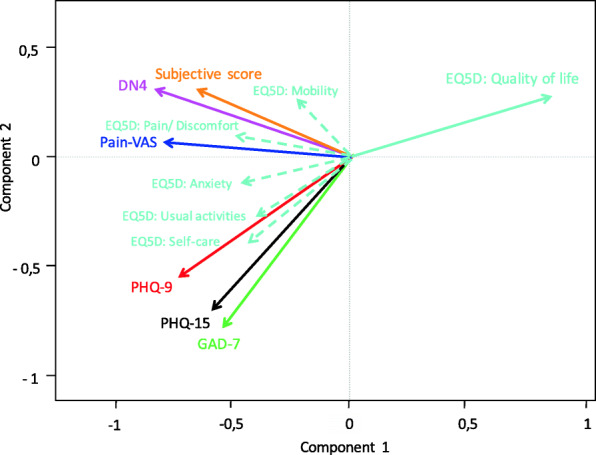
Biplot of subjective neurosensory measurements. The more similar the direction of two vectors, the stronger the correlation between these variables. A strong positive correlation suggests that the two questionnaires offer virtually the same information. This figure shows a negative correlation between quality of life and all other questionnaires. There is a strong positive correlation between Pain-VAS, DN4, and subjective score, as well as between PHQ-9, PHQ-15, and GAD-7 scores. Also, concerning the correlation between the individual questions for each questionnaire, those of the EQ5D-5 L scale where the least correlated with one another. However, these individual questions did correlate significantly with the other categorically related questionnaires, e.g., EQ5D-Pain correlated with DN4 and Pain-VAS, and EQ5D-Anxiety correlated with GAD-7 and PHQ-9 and -15. Therefore, the EQ5D-5 L can act as good screening questionnaire for assessing a patient’s subjective well-being

A biplot of all subjective measurements is shown in Fig. 4. The more similar the direction of two vectors, the stronger the correlation between the different questionnaires. A strong positive correlation suggests that the two questionnaires offered virtually the same information. Quality of life was negatively correlated with all other questionnaires. Pain-VAS, DN4, and subjective score project in similar directions toward the upper left quadrant, indicating a strong positive correlation among these measurements. The PHQ-9, PHQ-15, and GAD-7 questionnaire scores all project in similar directions toward the lower left quadrant, indicating a strong positive correlation among them. Also, concerning the correlation between the individual questions for each questionnaire, those of the EQ5D-5 L scale where the least correlated with one another and show the greatest scatter over the quadrants on the biplot. However, these individual subscales did correlate significantly with the other categorically related questionnaires, e.g., EQ5D-Pain correlated with DN4 and Pain-VAS, and EQ5D-Anxiety correlated with GAD-7 and PHQ-9 and -15. Therefore, these subscales of the EQ5D-5 L can act as good screenings for assessing a patient’s subjective well-being.

#### Objective and subjective measurements

**Fig. 5:**
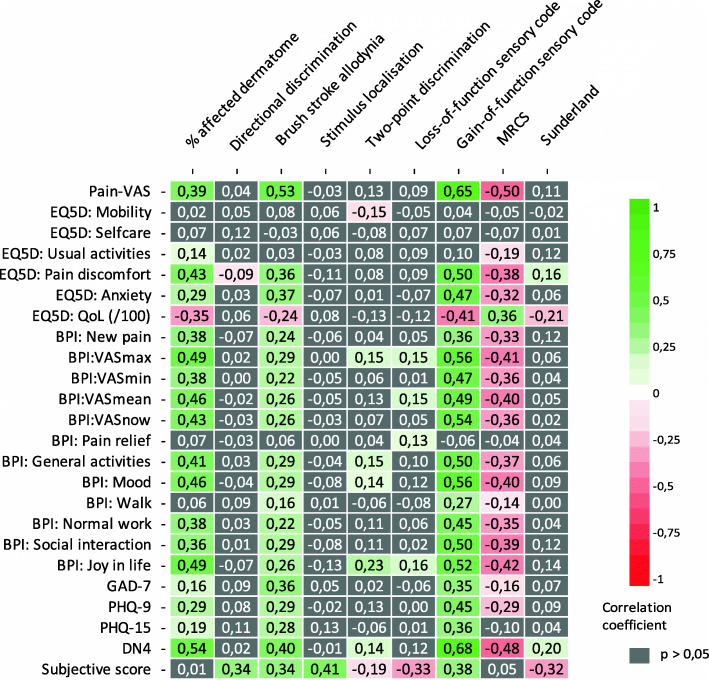
Correlation between objective (columns) and subjective (rows) neurosensory measurements. Correlation coefficients for significant positive correlations (*P* < 0.05) are shown in green. Correlation coefficients of significant negative correlations (*P* < 0.05) are shown in red. Non-significant correlations (*P* > 0.05) are shown in grey. This figure shows a pattern where generally the size of the affected area, the presence of brush stroke allodynia, and positive symptoms correlated with the different questionnaire scores e.g. Quality of life (EQ5D:QoL) correlated significantly with percentage of affected dermatome, brush stroke allodynia, gain-of-function sensory code, MRCS, and Sunderland. Pain-VAS, GAD-7, and PHQ-9 each correlate significantly with percentage of affected dermatome, brush stroke allodynia, gain-of-function sensory code, and MRCS. PHQ-15 correlated significantly with percentage of affected dermatome, brush stroke allodynia, and gain-of-function sensory code, but not with MRCS. The DN4 scores showed a significant correlation with percentage of affected dermatome, brush stroke allodynia, two-point discrimination, gain-of-function sensory code, MRCS, and Sunderland

Correlations between all objective and subjective measurements are shown in Fig. 5. As the figure shows, most of the objective neurosensory measurements did not correlate (*P* < 0.05) with the subjective questionnaires. Quality of life, however, correlated significantly with percentage of affected dermatome, the presence of brush stroke allodynia, gain-of-function sensory code, MRCS, and Sunderland. Quality of life did not correlate significantly with directional discrimination, stimulus localization, two-point discrimination, or loss-of-function sensory code. Pain-VAS, GAD-7, and PHQ-9 each correlated significantly with percentage of affected dermatome, brush stroke allodynia, gain-of-function sensory code, and MRCS. PHQ-15 correlated significantly with percentage of affected dermatome, brush stroke allodynia, and gain-of-function sensory code, but not with MRCS. The DN4 scores showed a significant correlation with percentage of affected dermatome, brush stroke allodynia, two-point discrimination, gain-of-function sensory code, MRCS, and Sunderland. The pattern is generally that the size of the affected area, presence of brush stroke allodynia, and positive symptoms correlated with the different questionnaire scores.

**Fig. 6:**
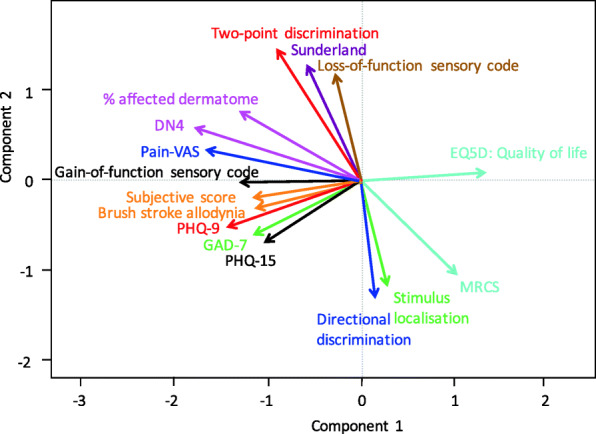
Biplot of all objective and subjective neurosensory measurements. An acute angle indicates a positive correlation. A 90-degree angle indicates no correlation between the two variables, and an obtuse angle indicates a negative correlation. There was a negative correlation of quality of life with gain-of-function sensory code, brush stroke allodynia, and percentage of affected dermatome. In addition, the other questionnaire scores (PHQ-15, GAD-7, PHQ-9, subjective score, Pain-VAS, and DN4) correlated positively with sensory gain-of-function, brush stroke allodynia, and percentage of affected dermatome. Little to no correlation was identified between the different questionnaire scores and the objective measurements of stimulus localization, directional discrimination, two-point discrimination, Sunderland score, and sensory loss-of-function

A biplot of all objective and subjective measurements is shown in Fig. 6. As noted, the more similar the direction of two vectors, the stronger the correlation between the variables. Quality of life negatively correlated with gain-of-function sensory code, brush stroke allodynia, and percentage of affected dermatome. In addition, the other questionnaire scores (PHQ-15, GAD-7, PHQ-9, subjective score, Pain-VAS, and DN4) positively correlated with gain-of-function sensory code, brush stroke allodynia, and percentage of affected dermatome. A poor to no correlation was found for each of the questionnaire scores and the objective measurements of stimulus localization, directional discrimination, two-point discrimination, Sunderland score, and loss-of-function sensory code.

### Prediction model

A prediction model for neurosensory recovery after 6 months of follow-up was constructed using baseline measurements and in accordance with the TRIPOD statement [30]. Criteria used to define near-to-complete recovery are shown in Supplemental Table S3. All criteria had to have been checked to qualify for a status of near-to-complete recovery. Details of the prediction model after 6 months are shown in Table 1.

**Table 1:** Prediction model for neurosensory recovery in PTN after 6 months

Variable	Coefficient	Confidence interval
Intercept	3.4109	[−1.4975; 8.3192]
Pain-VAS	0.048	[0.0079; 0.088]
% affected dermatome	−0.0316	[−0.071; 0.0078]
Sensory code Gain	−1.1032	[−2.3562; 0.1499]
2-point discrimination (affected side)	−0.1708	[−0.4153; 0.0737]

In the model, values for the pain visual analogue scale (Pain-VAS, 0–100), percentage of affected dermatome (0%–100%), gain-of-function sensory code (0–3), and two-point discrimination of the affected side (in mm) are multiplied by their corresponding coefficient. The resulting values are summed, and the intercept value is added to the total. For results ≥0, PTN is predicted to recover at 6 months. For values < 0, the PTN is predicated to not have recovered at 6 months

For the model, values for Pain-VAS (0–100), percentage of affected dermatome (0%–100%), gain-of-function sensory code (0–3), and two-point discrimination of the affected side (in mm) were multiplied by their corresponding coefficient. Then, these values were summed, and the intercept value was added to the total sum. If the result was greater than or equal to zero, the model predicted that the PTN will have resolved at 6 months. If the value was negative, the model predicted no PTN resolution after 6 months. The power of this association is illustrated in Table 2. When the model predicted no recovery after 6 months, chances of no recovery were high, for a negative predictive value of 87%. However, when the result was positive and thus predicted near-to-full recovery at 6 months, the positive predictive value was only 60%. Model sensitivity was 43%, and specificity was 93%.

**Table 2:** Power of the prediction model for neurosensory recovery in post-traumatic trigeminal neuropathy after 6 months

	Predicted recovery: No	Predicted recovery: Yes
Recovery: No	27	2
Recovery: Yes	4	3

Power of the prediction model for neurosensory recovery in PTN after 6 months. The model shows a negative predictive value of 87% and a positive predictive value of 60%

## Discussion

This study sought to answer the following three questions: Is there a correlation between the objective and subjective measurements? Which of these objective measurements has the greatest correlation with subjective well-being? Can we predict neurosensory outcome using baseline measurements?

We evaluated the correlation between clinical neurosensory tests and subjective questionnaires in patients with PTN who were followed and treated at the Department of Oral and Maxillofacial Surgery, University Hospital Leuven, during a 2-year period. Both types of information were collected simultaneously on multiple occasions during an average follow-up of 566 days.

Demographics of the study population (age, sex, cause of injury, affected division of trigeminal nerve, etc.) are similar to what others have described previously [2, 11, 13, 15, 23, 27, 31, 32] and discussing these findings as such would be beyond the scope of this paper. Similarly, the evolution of separate clinical neurosensory tests or individual subjective assessments would likely interest only researchers evaluating specific interventions in PTN. We do want to mention, however, the difficulty of objectively declaring a clinical neurosensory examination as “improved” given that improvement might be of little value for the patient, and identical clinical examinations could even be perceived differently. Furthermore, in the process of neurosensory recovery, positive symptomatology can arise, leaving the patient in a potentially worse situation. It is therefore important that we understand the correlation between these clinical neurosensory tests and patient’s subjective well-being.

### Correlation analysis

We found a statistically significant correlation (*P* < 0.05) between subjective well-being and some aspects of the clinical neurosensory evaluation. When neuropathy presented with brush stroke allodynia, mechanical or thermal hyperesthesia, or a large zone size, the effect on the patient’s subjective well-being is expected to be substantial. In contrast, limited two-point discrimination, inability to determine direction of movement or locate a stimulus in a compromised dermatome were not significantly correlated with self-assessed well-being. Although both positive and negative symptomatology can co-exist in PTN, these results do suggest that positive symptoms dominate the effect on affect.

Only a handful of studies have compared the relation between objective and subjective data in PTN. Pogrel [33] found that semi-objective assessment of patients does not always correspond with the patient’s subjective evaluation. Shintani et al. [34] found no evidence of an association between subjective and objective symptoms after lingual nerve repair. In contrast, Susarla et al. [35] described a strong correlation in this regard. In their study, patients who experienced greater neurosensory improvement reported lower frequencies of related oral dysfunction.

Furthermore, higher scores for pain-VAS, subjective functioning, GAD-7, PHQ-9, PHQ-15, and the DN4 questionnaire all correlated significantly with a poorer quality of life and with one another in the current work. These results are in accordance with past observations of an association of depression and anxiety with somatic symptoms [36–38] and more severe pain with elevated levels of depression, pain catastrophizing, and reduced quality of life and coping efficacy levels [15, 24].

This also suggest that the routine use of multiple validated questionnaires in daily practice provides little additional information in comparison to using only one or two questionnaires to assess patient subjective well-being. We found the EQ5D-5 L scale to be the most clinically useful because it is short and its individual questions each provide mainly new information.

Nevertheless, managing PTN requires a holistic approach with sufficient attention to psychosocial well-being. It is the combination of environmental, psychosocial, and genetic factors that cause identical injuries to produce a large variability in PTN [39–41]. In addition, improvement on qualitative sensory testing cannot be viewed as successful if the patient is still suffering from other debilitating symptoms [19]. Furthermore, in patients reporting poor subjective well-being in the absence of positive symptoms or a large neuropathic area, additional attention towards psychosocial triggers might enhance treatment outcome.

### Prediction model

To our knowledge, this study is the first to propose a clinical prediction model using baseline clinical neurosensory test values to give an indication of expected neurosensory recovery in patients with PTN. A negative predictive value of 87% for 6 months of follow-up was found. The positive predictive value of the model was quite limited, however. Whether this model can be validated in future studies remains to be seen, but if so, it could contribute to establishing realistic expectations about the likelihood of neurosensory recovery.

### Limitations

The study was conducted at a single referral center. Also, case-wise deletion excluded nine patients because of missing data. Furthermore, observer bias is possible because only one observer (FVDC) saw all patients. This bias is, however, somewhat controlled by the standardized protocol that was used. Similar studies can be performed in larger samples or other referral centers to evaluate the validity of the prediction model and the observed correlations.

### Conclusion

We found a statistically significant correlation between subjective well-being and brush stroke allodynia, mechanical or thermal hyperesthesia, and the size of the neuropathic area in patients with PTN. No significant correlation was found for two-point discrimination, directional discrimination, stimulus localization, or sensory loss-of-function phenotype.

## Supplementary Information

**Additional file 1: Table S1.** Medical Research Council Scale for sensory recovery. **Figure S1.** Sunderland Clinical classification system (Miloro Modification). **Table S2.** Patient characteristics table. **Figure S2.** Distribution of cases by Mechanism. **Figure S3.** Distribution of PTN cases by MRCS-score at baseline vs at final follow-up moment. **Figure S4.** Distribution of PTN cases by Sunderland Clinical classification score at baseline vs at final follow-up moment. **Figure S5.** Distribution of PTN cases by sensory phenotype at baseline vs at final follow-up. **Figure S6.** Evolution of subjective functioning in PTN. **Table S3.** Criteria for near to complete neurosensory recovery in PTN.

## Data Availability

The database and R-script used and/or analyzed during the current study are available from the corresponding author on reasonable request. Literature included in this study is available through the relevant journals.

## Abbreviations

PTN: Post-traumatic trigeminal neuropathy
LN: Lingual nerve
IAN: Inferior alveolar nerve
ICOP: International classification of orofacial pain
MRCS: Medical research council scale
EQ5D-5L: EuroQol 5 dimension scale
GAD-7: General anxiety disorder-7
PHQ-9: Public health questionnaire 9
PHQ-15: Public health questionnaire 15
DN4: Douleur Neuropathique 4
BPI: Brief pain inventory
VAS: Visual analogue scale
QoL: Quality of life
