# Supplementary material for: Post-traumatic trigeminal neuropathy: correlation between objective and subjective assessments and a prediction model for neurosensory recovery
Source: J Headache Pain. 2021 May 24;22(1):44. doi: 10.1186/s10194-021-01261-3 (PMC8146662; doi:10.1186/s10194-021-01261-3)
Supplement: Supplementary file 1 — Additional file 1: Table S1. Medical Research Council Scale for sensory recovery. Figure S1. Sunderland Clinical classification system (Miloro Modification). Table S2. Patient characteristics table. Figure S2. Distribution of cases by Mechanism. Figure S3. Distribution of PTN cases by MRCS-score at baseline vs at final follow-up moment. Figure S4. Distribution of PTN cases by Sunderland Clinical classification score at baseline vs at final follow-up moment. Figure S5. Distribution of PTN cases by sensory phenotype at baseline vs at final follow-up. Figure S6. Evolution of subjective functioning in PTN. Table S3. Criteria for near to complete neurosensory recovery in PTN. [file 10194_2021_1261_MOESM1_ESM.docx]

**Supplemental material**

**Meewis et al. (2021) Post-traumatic trigeminal neuropathy: correlation between objective and subjective assessments and a prediction model for neurosensory recovery**

Table S1. Medical Research Council Scale for sensory recovery

Figure S1. Sunderland Clinical classification system (Miloro Modification)

Table S2. Patient characteristics table

Figure S2. Distribution of cases by Mechanism

Figure S3. Distribution of PTN cases by MRCS-score at baseline vs at final follow-up moment

Figure S4. Distribution of PTN cases by Sunderland Clinical classification score at baseline vs at final follow-up moment

Figure S5. Distribution of PTN cases by sensory phenotype at baseline vs at final follow-up

Figure S6: Evolution of subjective functioning in PTN

Table S3. Criteria for near to complete neurosensory recovery in PTN

**Table S1. Medical Research Council Scale for sensory recovery.**

| **Table S1** |  |
| --- | --- |
| **Medical Research Council Scale for sensory recovery*** | |
| S0: Absence of sensibility in the autonomous area of the nerve | |
| S1: Recovery of deep cutaneous pain and tactile sensibility | |
| S1+: Recovery of superficial pain sensibility | |
| S2: Recovery of some degree of superficial cutaneous pain and tactile sensibility | |
| S2+: As in S2, but with overresponse | |
| S3: Return of pain and tactile sensibility with disappearance of overresponse, s2PD>15mm, m2PD>7mm | |
| S3+: Return of sensibility as in S3 with some recovery of 2-point discrimination, s2PD: 7-15mm, m2PD: 4-7mm | |
| S4: Complete recovery, s2PD: 2-6mm, m2PD: 2-3mm | |
| * Birch R, Bonney G, Wynn-Parry CB. Surgical Disorders of the Peripheral Nerves. Philadelphia: Surg. 1992;30(6):387-389. | |

**Figure S1. Sunderland Clinical classification system (Miloro Modification)**

| **Table S2** | |  | | | | | | | | |
| --- | --- | --- | --- | --- | --- | --- | --- | --- | --- | --- |
| **Patient characteristics table** | | | | | | | | | | |
| Total | | | | n | | | | | 36 | |
| Age | | | | Years (SD, range) | | | | | 42 (12.5, 23-68) | |
| Gender | | | | 23 female | | | | | 13 male | |
| Time since injury | | | | Days (SD, range) | | | | | 210 (289, 3-1073) | |
| Follow-up period | | | |  |  |  |  |  | 566 (218, 149-865) | |
|  | | | | | | | | | **n (%)** | |
| Mechanism of injury | | | Third molar removal | | | | | | 17 (47) | |
|  |  |  | Implant placement | | | | | | 4 (11) | |
|  |  |  | Facial trauma | | | | | | 4 (11) | |
|  |  |  | Local anesthesia | | | | | | 3 (8) | |
|  |  |  | Non-wisdom tooth extraction | | | | | | 2 (6) | |
|  |  |  | Endodontic treatment | | | | | | 2 (6) | |
|  |  |  | Other | | | | | | 5 (14) | |
| Site of injury | | | Inferior alveolar nerve | | | | | | 23 (64) | |
|  |  |  | Lingual nerve | | | | | | 10 (28) | |
|  |  |  | Maxillary nerve | | | | | | 7 (19) | |
|  |  |  | Ophthalmic nerve | | | | | | 1 (3) | |
|  |  |  | Right-sided PTN | | | | | | 19 (53) | |
|  |  |  | Left-sided PTN | | | | | | 17(47) | |
| QST | | | Total | | | | | | 5 (14) | |
| Surgical reintervention | | | Total | | | | | | 7 (19) | |
|  |  |  | Buccal fat wrapping | | | | | | 5 (14) | |
|  |  |  | Microsurgical repair | | | | | | 2 (6) | |
|  |  |  | Decompression | | | | | | 2 (6) | |
|  |  |  | Neuroma excision | | | | | | 2 (6) | |
|  |  |  | Foreign body removal | | | | | | 1 (3) | |
| MRN | | | Total | | | | | | 7 (19) | |
|  |  |  | Lead to change of policy | | | | | | 5 (71) | |
|  |  |  | Lead to surgical reintervention | | | | | | 3 (43) | |
| Reported symptoms | | | Numbness | | | | | | 31 (86) | |
|  |  |  | Pain | | | | | | 16 (44) | |
|  |  |  | Stinging pain | | | | | | 11 (31) | |
|  |  |  | Nagging | | | | | | 10 (28) | |
|  |  |  | Burning | | | | | | 10 (28) | |
|  |  |  | Sensitive | | | | | | 10 (28) | |
|  |  |  | Swollen | | | | | | 10 (28) | |
|  |  |  | Stinging | | | | | | 7 (19) | |
|  |  |  | Pulling | | | | | | 7 (19) | |
|  |  |  | Electrical | | | | | | 6 (17) | |
|  |  |  | Tickling | | | | | | 6 (17) | |
|  | | | | | | | | | | |
| **Neurosensory assessment** | | | | | | | | | **Baseline** | **Final follow-up** |
| Percentage of affected dermatome | | | | | | | % (SD) | | 91 (21) | 40 (46) |
| Two-point discrimination | | | | | | | mm (SD) | | 14 (7) | 8 (5) |
| Stimulus localization | | | | | | | /5 (SD) | | 3 (1) | 4 (2) |
| Directional discrimination | | | | | | | /10 (SD) | | 6 (3) | 9 (3) |
| Brush stroke allodynia | | | | | | | n (%) | | 11 (31) | 5 (14) |
| MRCS | | | S0 | | | | n (%) | | 5 (14) | 0 (0) |
|  |  |  | S1 | | | |  |  | 0 (0) | 0 (0) |
|  |  |  | S2 | | | |  |  | 1 (3) | 0 (0) |
|  |  |  | S2+ | | | |  |  | 10 (28) | 5 (14) |
|  |  |  | S3 | | | |  |  | 8 (22) | 1 (3) |
|  |  |  | S3+ | | | |  |  | 11 (31) | 7 (19) |
|  |  |  | S4 | | | |  |  | 1 (3) | 23 (64) |
| Sunderland | | | V | | | | n (%) | | 5 (14) | 0 (0) |
|  |  |  | IV | | | |  |  | 11 (31) | 6 (17) |
|  |  |  | III | | | |  |  | 5 (14) | 2 (6) |
|  |  |  | II | | | |  |  | 5 (14) | 3 (8) |
|  |  |  | I | | | |  |  | 10 (28) | 25 (69) |
| Sensory phenotype | | | Loss of function | | | L0 | n (%) | | 4 (11) | 24 (67) |
|  |  |  |  |  |  | L1 |  |  | 1 (3) | 2 (6) |
|  |  |  |  |  |  | L2 |  |  | 9 (25) | 2 (6) |
|  |  |  |  |  |  | L3 |  |  | 22 (61) | 8 (22) |
|  |  |  | Gain of function | | | G0 | n (%) | | 19 (53) | 28 (78) |
|  |  |  |  |  |  | G1 |  |  | 1 (3) | 2 (6) |
|  |  |  |  |  |  | G2 |  |  | 5 (14) | 3 (8) |
|  |  |  |  |  |  | G3 |  |  | 11 (31) | 3 (8) |
| Pain  (pain-VAS) | | |  | | | | /100 (SD) | | 20 (26) | 13 (25) |
| Quality of life  (EQ5D) | | | Mobility | | | | → ≥ 3/5, % | | 1 (3) | 1 (3) |
|  |  |  | Selfcare | | | |  |  | 0 (0) | 0 (0) |
|  |  |  | Daily activities | | | |  |  | 3 (8) | 0 (0) |
|  |  |  | Discomfort | | | |  |  | 5 (14) | 3 (8) |
|  |  |  | Anxiety | | | |  |  | 1 (3) | 0 (0) |
|  |  |  | VAS Quality of Life | | | | /100 (SD) | | 59 (18) | 72 (22) |
| Anxiety  (GAD-7) | | | Severe | | | | n (%) | | 7 (11) | 3 (8%) |
|  |  |  | Moderate | | | |  |  | 1 (3) | 2 (5) |
|  |  |  | Mild | | | |  |  | 15 (42) | 8 (22) |
| Depression  (PHQ-9) | | | Severe | | | | n (%) | | 2 (6) | 4 (11) |
|  |  |  | Moderately severe | | | |  |  | 2 (6) | 3 (8) |
|  |  |  | Moderate | | | |  |  | 5 (14) | 1 (3) |
|  |  |  | Mild | | | |  |  | 15 (42) | 8 (22) |
| Somatic severity of symptoms  (PHQ-15) | | | High | | | | n (%) | | 4 (11) | 2 (6) |
|  |  |  | Medium | | | |  |  | 11 (31) | 7 (19) |
|  |  |  | Low | | | |  |  | 8 (22) | 9 (25) |
| Neuropathic pain  (DN4) | | |  | | | | → ≥ 3/7, % | | 18 (50) | 10 (28) |
| BPI | Pain severity | | | | Worst pain in past 24h | | → ≥ 3/10, % | | 22 (61) | 11 (31) |
|  |  |  |  |  | Least pain in past 24h | |  |  | 12 (33) | 7 (19) |
|  |  |  |  |  | Average pain | |  |  | 21 (58) | 9 (25) |
|  |  |  |  |  | Pain now | |  |  | 17 (47) | 7 (19) |
|  |  | | | |  | | | |  | |
|  | Pain medication | | | |  | | | | Number of prescriptions | |
|  |  |  |  |  | Corticosteroids | | | | 27 | |
|  |  |  |  |  | Vitamin B IM | | | | 26 | |
|  |  |  |  |  | Vitamin B per os | | | | 28 | |
|  |  |  |  |  | Acetaminophen | | | | 6 | |
|  |  |  |  |  | Ibuprofen | | | | 21 | |
|  |  |  |  |  | Amitriptyline | | | | 4 | |
|  |  |  |  |  | Baclofen | | | | 1 | |
|  |  |  |  |  | Pregabalin | | | | 4 | |
|  |  |  |  |  | Oxcarbazepine | | | | 1 | |
|  |  |  |  |  | Carbamazepine | | | | 2 | |
|  |  |  |  |  | Duloxetine | | | | 1 | |
|  |  |  |  |  | other | | | | 2 | |
|  |  |  |  |  | Percentage of relief provided in past 24h (SD) | | | | 41 (40) | 28 (41) |
|  | Pain interference | | | | General activities | | | → ≥ 3/10, % | 17 (47) | 7 (19) |
|  |  |  |  |  | Mood | | |  | 15 (42) | 9 (25) |
|  |  |  |  |  | Walking ability | | |  | 8 (19) | 2 (6) |
|  |  |  |  |  | Normal Tasks | | |  | 14 (39) | 9 (25) |
|  |  |  |  |  | Social interaction | | |  | 12 (33) | 5 (14) |
|  |  |  |  |  | Joy in life | | |  | 15 (42) | 8 (22) |
| Subjective score (SD) | | | | | | | | | 8 (4) | 10 (5) |
| QST = quantitative sensory tests; MRN = magnetic resonance imaging; MRCS = Medical research council scale for sensory recovery; EQ5D = EuroQol five-dimension scale; GAD-7 = General Anxiety Disorder questionnaire; PHQ-9 & PHQ-15 = Public Health questionnaire 9 and 15; DN4 = Douleur Neuropathique 4 questionnaire | | | | | | | | | | |

**Figure S2. Distribution of PTN cases by mechanism of injury.**

**Figure S3. Distribution of PTN cases by MRCS-score at baseline vs. at final follow-up moment.**

Figure S3: MRCS-score at baseline vs. at final follow-up moment. S0 = Absence of sensibility in the autonomous area of the nerve. S1 = Recovery of deep cutaneous pain and tactile sensibility. S1+ = Recovery of superficial pain sensibility. S2 = Recovery of some degree of superficial cutaneous pain and tactile sensibility. S2+ = as in S2, but with overresponse. S3 = Return of pain and tactile sensibility with disappearance of overresponse, s2PD>15mm, m2PD>7mm. S3+ = Return of sensibility as in S3 with some recovery of two-point discrimination, s2PD: 7-15mm, m2PD: 4-7mm. S4 = complete recovery, s2PD: 2-6mm, m2PD: 2-3mm.

**Figure S4. Distribution of PTN cases by Sunderland Clinical classification score at baseline vs. at final follow-up moment.**

Figure S4. Sunderland Clinical classification score at baseline vs. at final follow-up moment. Scoring system was applied as illustrated in figure S1.

**Figure S5. Distribution of PTN cases by sensory phenotype at baseline vs at final follow-up.**

Figure S5. Distribution of PTN cases by sensory phenotype at baseline vs at final follow-up moment. A code for sensory phenotype was assigned to each individual. All codes consist of a letter L (Loss of function or sensory deficit) and a letter G (Gain of function or hyperesthesia), followed by number 0 (none), 1 (thermal), 2 (mechanical) or 3 (mixed). For example, L3G0 stands for a patient with mixed sensory loss and no mechanical or thermal hyperesthesia.

**Figure S6: Evolution of subjective functioning in PTN.**

Figure S6: Evolution of subjective functioning in PTN. Patients were asked to score their current subjective function, ranging from 0 (complete anesthesia) to 20 (20 for the worst pain imaginable). A score of 10 would mean a normal function and no deficit. This figure shows trends for both positive and negative symptoms evolve in the same direction towards self-perceived normal functioning. A small group of outliers fail to return to this undisrupted status.

**Table S3. Criteria for near to complete neurosensory recovery**

| **Table S3** |  | |
| --- | --- | --- |
| **Post-traumatic trigeminal neuropathy: Criteria* to define near to complete neurosensory recovery** | | |
| **Variable** | | **Criterium** |
| % affected dermatome | | ≤ 10% |
| VAS | | ≤ 10/100 |
| Directional discrimination | | ≥ 9/10 |
| Brush stroke allodynia | | 0 |
| Stimulus localization | | ≥ 4/5 |
| Two-point discrimination | | ≤ 3mm |
| (affected - control side) | |  |
| Sensory phenotype Loss of function | | 0 |
| Sensory phenotype Gain of function | | 0 |
| MRCS | | S3+ or S4 |
| Sunderland | | I or II |
| *All criteria must be checked in order to qualify for a status of near to complete recovery | | |
